# Administration of oral fluoroquinolone and the risk of rhegmatogenous retinal detachment: A nationwide population-based study in Korea

**DOI:** 10.1371/journal.pone.0195563

**Published:** 2018-04-12

**Authors:** Seung Yong Choi, Hyun-A. Lim, Hyeon Woo Yim, Young-Hoon Park

**Affiliations:** 1 Department of Ophthalmology and Visual Science, College of Medicine, The Catholic University of Korea, Seoul, Republic of Korea; 2 Department of Preventive Medicine, College of Medicine, The Catholic University of Korea, Seoul, Republic of Korea; 3 Catholic Institute for Visual Science, College of Medicine, The Catholic University of Korea, Seoul, Republic of Korea; National Yang-Ming University Hospital, TAIWAN

## Abstract

**Purpose:**

To investigate the association between oral fluoroquinolones (FQ) and the risk of rhegmatogenous retinal detachment (RRD) using a nationwide population-based study in Korea, designed to control for time-related bias.

**Methods:**

As a nested case-control study within a cohort, the KNHIS-NSC 2002–2013 (Korean National Health Insurance Service National Sample Cohort) data used for the investigation. The subjects who visited an ophthalmologist were included in a cohort. Subjects with infectious ocular diseases, severe ocular trauma, and congenital diseases were excluded. Within the cohort, subjects who underwent surgery for RRD were defined as cases, and controls were matched by age group, sex, and cohort entry date using an incidence density sampling method. After investigating the exposure to oral FQ, the odds ratio was calculated by the FQ exposure rate of both groups and adjusted by the confounding factors of demography, health service utilization, and comorbidities.

**Results:**

A total of 1,151 subjects in the case group and 11,470 subjects in the control group were included. There were intergroup differences in household income, numbers of ophthalmologic visits and drug prescriptions, events of intraocular surgeries, and prevalence of diabetes and degenerative myopia (all P’s<0.05). The crude odds ratio of the total group was 1.06 (P = 0.53, 95% CI 0.88–1.28), and the odds ratio adjusted for all pre-defined confounders was 1.00 (P = 0.99, 95% CI 0.81–1.24). The crude and adjusted odds ratios were not showed statistical significance (all P’s>0.05).

**Conclusions:**

By the nested case-control design, this study showed that oral administration of FQ was not associated with the increased risk of development of RRD.

## Introduction

Fluoroquinolones (FQ) are a commonly prescribed class of antibiotics and the recommended drugs for patients with acute bacterial infection of the respiratory and urinary tract [1]. Some reports have suggested the possibility of retinal toxicity from FQ, such as retinal hemorrhage [2], retinal degeneration [3], and retinal detachment (RD) [4,5]. Recently, several population-based studies showed a relationship between administration of oral FQ and development of retinal detachment [6–9].

There is no proven mechanism that might explain the development of RD after FQ. The effect of FQ on collagen fibers in the vitreous is a possible mechanism for the increased risk of RD after administration of oral FQ [6,7]. However, other population-based studies have suggested that there is no relationship between development of RD and use of FQ [10–13]. Because of the limited evidence for the underlying mechanism and conflicting results from population-based studies, it is difficult to conclude that FQ use increases the risk of development of RD.

Compared with randomized clinical trials, a cohort or case-control study design that uses population-based data is a more effective tool for studying rare adverse events of drugs [14]. However, the potential risk of bias and confounding factors can affect the results of a population-based study. Using an inappropriate method for case selection can result in selection bias [15]. In studies associated with drug exposure, time-related biases including immortal bias and time-window bias are potential factors that can limit interpretation of the results [16–18].

In this study, we investigated the association between oral FQ and the risk of rhegmatogenous retinal detachment (RRD) using a nationwide population-based study in Korea, using a nested case-control design that could minimize the biases reported in drug exposure studies.

## Methods

### Statement of ethics

The authors investigated this study under the approval of the Institutional Review Board of Seoul St. Mary’s Hospital, College of Medicine, The Catholic University of Korea (IRB#KC17ZESI1449), and respected the tenets of the Declaration of Helsinki. The author performed the investigation without requirement for informed consent, because all data used in this study were fully anonymized before it was provided to the authors.

### Materials

Most Koreans are registered in the Korean National Health Insurance Service (KNHIS) database [19]. The KNHIS database includes data for demographic factors (age group, sex, household income, and residential area) and medical factors (diagnosis, history of procedures or surgeries, and drug prescriptions). Approximately one million subjects (2% of total data) were randomly selected and released as the National Sample Cohort (NSC) [19]. In this study, we used the KNHIS-NSC 2002–2013, which includes the medical service records of subjects in the cohort from January 2002 to December 2013.

### Study design and cohort setting

For the design of a nested case-control study within a nested cohort, we set up a nested cohort of patients who visited an ophthalmologist at least once from 2002 to 2013. Using the data in the KNHIS-NSC 2002–2013, we defined a slit-lamp microscope examination as an ophthalmologic visit and searched for the EDI (electronic data interchange) codes for a slit-lamp microscope (E6810, EY791, EY792, or EY799) for the target setting of the cohort.

History of infectious endophthalmitis, infectious chorioretinitis, ocular trauma requiring surgical intervention, and congenital diseases were exclusion criteria. Subjects with exclusion criteria were identified by searching the KCD-6 (Korean standard Classification of Diseases, 6th revision) codes, which corresponded with the same code in the ICD-10 (International Classification of Diseases, 10th Revision). Subjects with ICD-10 codes for infectious endophthalmitis (H44.0, H44.1, and H45.1), intraocular foreign body (H44.6 and H44.7), infectious chorioretinitis (H32.0 and A18.5 for tuberculosis, B58.0 for toxoplasma, A52.7 for syphilis, and B25.8 for cytomegalovirus), blow-out fracture (S02.1, S02.3, and S02.8), penetrated ocular trauma (S05.2, S05.3, S05.4, S05.5, and S05.6), and congenital diseases (any code starting with Q, from Q00 to Q99) were excluded.

For the subjects in the nested cohort, the earliest day of an ophthalmologic visit was defined as the cohort entry date. Enrolled subjects were followed up from the cohort entry date to the date of surgery for RRD, death, or the last day of the KNHIS-NSC 2002–2013 (December 31, 2013). Follow-up durations were calculated from the cohort entry date to the last follow-up date.

### Definitions of a case and control matching

A case was defined as a patient who had a RRD with a record of a surgery for RRD (scleral buckling surgery or pars plana vitrectomy) in a nested cohort. For selection of a case, we searched subjects who had ICD-10 codes of RRD (H33.0, H33.4, and H33.5) as a main diagnostic code and an EDI code of surgery for RRD (S5130 for scleral buckling surgery or S5121 for pars plana vitrectomy). Subjects who had an EDI code of S5122 (partial vitrectomy), ICD-10 codes of exudative RD (H33.2) and tractional RD (H33.4) were not included. An additional exclusion was done for subjects who were diagnosed with retinal detachment without surgery at the cohort entry date. The first date with diagnostic code of RRD was defined as an index date.

A control was defined as a patient who did not undergo surgery for RRD in a nested cohort. Control matching at a 1:10 ratio was performed by an incidence density sampling method. Age group at cohort entry, sex, and the time of cohort entry were selected as matching factors. An index date of matching controls was defined as an index date of a matched case.

### Exposure and confounders

From 365 days before the index date to the index date, cases were searched for prescriptions for any oral FQ. Prescriptions for oral FQ in the matching controls were also investigated over the same duration. The subjects with prescriptions for oral FQ in ophthalmology cases were excluded (prescriptions for oral FQ with ICD-10 codes from H00 to H59, which were classified as diseases of the eye and adnexa). Because of unreported association with RRD development, the exposure to topical FQ was excluded. The 18 ingredients of oral FQ including ciprofloxacin, levofloxacin, and moxifloxacin were investigated by the main ingredient code in the KNHIS-NSC data. Certain ingredients and their main ingredient codes are listed in S1 Table.

The group with exposure was divided into 5 subgroups based on the interval between the index date and day of exposure to FQ: groups with 0–14, 15–90, 91–180, 181–270, and 271–365 days after exposure (subgroup 1, 2, 3, 4, and 5, respectively). For subjects with multiple prescriptions for FQ, the most recent day of the prescription was selected as the date of exposure.

Factors that could have associations with health service accessibility, severity of ocular disease, or development of RRD were investigated as confounding factors: household income, residential area, numbers of ophthalmologic visits and prescribed drugs, follow-up duration in a cohort, diabetes, degenerative myopia, cataract surgery, and vitrectomy for diseases other than RRD. Subjects with diabetes were identified by the ICD-10 codes of E10-E14 and H36.0, and subjects with degenerative myopia were identified by the ICD-10 code of H44.2. Cataract surgery was identified by the surgical codes for phacoemulsification (S5119), extracapsular or intracapsular lens extraction (S5111), and primary intraocular lens implantation (S5117). Glaucoma surgery was identified by the codes for filtering surgery (S5042) and drainage implant surgery (S5049). Corneal transplantation was identified by the codes for penetrating keratoplasty (S5372). All subjects who had an EDI code of pars plana vitrectomy (S5121) without a main diagnostic code of RRD were also investigated for exclusion of confounding factors.

### Statistical analysis

For the characteristics of the cases and controls, nominal-level variables were compared by the chi-square test, and metric variables were compared by Student’s t-test and Fisher’s exact test. Exposure rates were measured in both cases and controls, and the odds ratio and 95% CI (confidence interval) were calculated using conditional logistic regression [20]. The adjusted odds ratio was investigated after correction for confounding factors. The statistical analysis software SAS (version 9.4, SAS Inc, Cary, NC, USA) was used for all study processes and statistical analysis.

## Results

**Fig 1:**
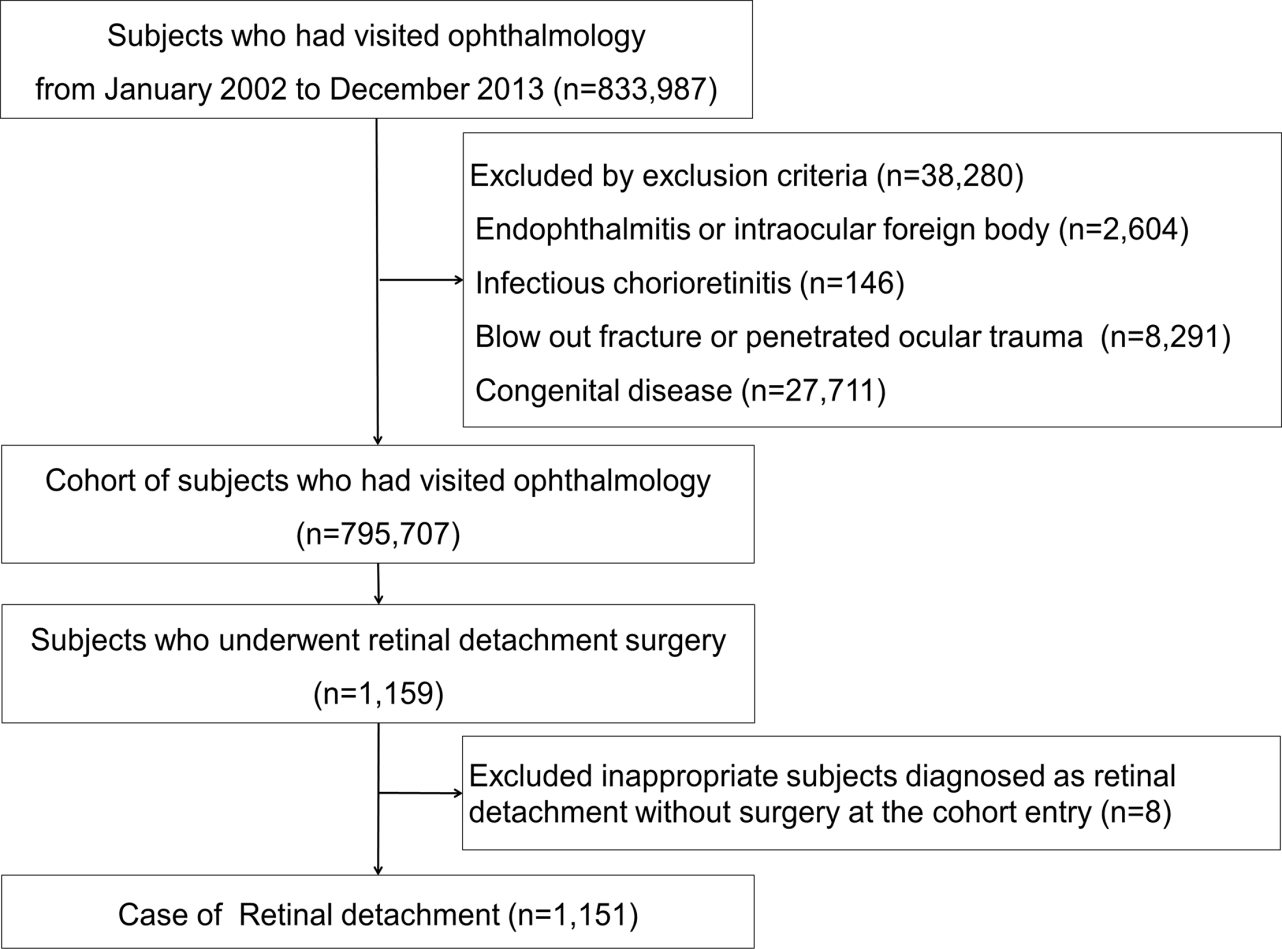
Flow chart showing the case search process. From a total of 833,987 subjects, 795,707 were included in a cohort after applying the exclusion criteria. The final case set included 1,151 subjects from the total 1,159 subjects who underwent retinal detachment surgery.

Out of 1,025,340 subjects in the KNHIS-NSC 2002–2013 database, there were 833,983 subjects who had visited an ophthalmologist from January 2002 to December 2013. A cohort of 795,707 subjects was identified after excluding 38,280 subjects with exclusion criteria. Of 1,159 subjects who underwent RD surgery, 8 who were diagnosed with retinal detachment without surgery at the cohort entry date were excluded. The final case set included 1,151 subjects, and the matching control group included 11,470 subjects. Fig 1 shows a flow chart of the case search process.

In terms of control matching factors, there were no differences in the distribution of age groups at cohort entry date or sex (P = 0.99, P = 0.98, respectively). The mean duration of follow up in the cohort, the other matching factor, was 3.2±3.2 years in the case group and 3.2±3.2 years in the control group, without a intergroup difference (P = 0.91). The distribution of residential areas did not show differences (P = 0.34), but household income showed different distributions between the case and control groups (P = 0.004) (Table 1).

**Table 1:** Comparisons of characteristics and comorbidities between the case and control groups.

	Case (n = 1151)	Control (n = 11470)	*P-*value
**Demography**			
Age group at cohort entry			0.99
0–9	22 (1.9%)	220 (1.9%)	
10–19	85 (7.4%)	850 (7.4%)	
20–29	135 (11.7%)	1346 (11.7%)	
30–39	136 (11.8%)	1359 (11.9%)	
40–49	215 (18.7%)	2150 (18.7%)	
50–59	272 (23.6%)	2714 (23.7%)	
60–69	211 (18.3%)	2092 (18.2%)	
70–79	63 (5.5%)	619 (5.4%)	
≥80	12 (1.0%)	120 (1.1%)	
Sex			0.98
Male	649 (56.4%)	6472 (56.4%)	
Female	502 (43.6%)	4998 (43.6%)	
Residential area			0.34
Capital area	568 (49.4%)	5525 (48.2%)	
Other urban area	248 (21.6%)	2370 (20.7%)	
Rural area	335 (29.1%)	3575 (31.2%)	
Household income			0.004
Decile 0–2	154 (13.4%)	1609 (14.0%)	
Decile 3–5	268 (23.3%)	2717 (23.7%)	
Decile 6–8	331 (28.8%)	3737 (32.6%)	
Decile 9–10	398 (34.6%)	3407 (29.7%)	
Follow up duration (years, mean ± SD)	3.2 ± 3.2	3.2 ± 3.2	0.91
**Health services during the year prior to the index date**			
Ophthalmologic visits	943 (81.9%)	6561 (57.2%)	<0.001
Number of visits	3.8 ± 3.9	1.9 ± 2.1	<0.001
Drug prescriptions	984 (85.5%)	10272 (89.6%)	<0.001
Number of prescribed drugs	11.0 ± 11.2	8.6 ± 11.1	<0.001
**Comorbidities during the year prior to the index date**			
Cataract surgery	73 (6.3%)	142 (1.2%)	<0.001
Glaucoma surgery	1 (<0.1%)	0 (0%)	0.09*
Keratoplasty	1 (<0.1%)	0 (0%)	0.09*
Vitrectomy	41 (3.6%)	8 (0.1%)	<0.001*
Diabetes	204 (17.7%)	1133 (9.9%)	<0.001
Degenerative myopia	4 (0.4%)	6 (0.1%)	0.001*

*Fisher’s exact test

The percentage of subjects undergoing ophthalmologic visits for one year prior to the index date was larger in the case group (943 subjects, 81.9%) than in the control group (6,561 subjects, 57.2%) (P<0.001). For the subjects undergoing ophthalmologic visits, the mean number of visits was larger in the case group (3.8±3.9) than in the control group (1.9±2.1) (P<0.001). The percentage of subjects with drug prescriptions at any clinic on the one year prior to the index date was lower in the case group (984 subjects, 85.5%) compared to the control group (10,272 subjects, 89.6%) (P<0.001). For the subjects with drug prescriptions, there was a larger mean number of prescriptions in the case group (11.0±11.2) than in the control group (8.6±11.1) (P<0.001) (Table 1).

Comorbidities that were associated with incidence of RD were more frequent in the case group than in the control group for the year prior to the index date. In the case and control groups, the percentage of subjects who underwent cataract surgery was 6.3% (73 subjects) and 1.2% (142 subjects), respectively (P<0.001). There was one subject each who underwent glaucoma filtering surgery and penetrating keratoplasty in a case group. Any subject underwent glaucoma surgery or keratoplasty in a control group (all P’s = 0.09). The percentage of subjects who underwent vitrectomy not for treatment of RD was also higher in the case group (41 subjects, 3.6%) than in the control group (8 subject, 0.1%) (P<0.0001, Fisher’s exact test). The case group also had a higher incidence of diabetes (204 subjects, 17.7%) than the control group (1,133 subjects, 9.9%) (P<0.001). There were 4 (0.4%) and 6 subjects (0.1%) subjects with degenerative myopia in the case and control groups, respectively, representing a statistical difference (P = 0.01, Fisher’s exact test) (Table 1).

There were 146 (12.7%) and 1,384 (12.1%) subjects with exposure to oral FQ in the year prior to the index date in the case and control groups, respectively. Of the 18 ingredients used for exposure assessment, the case group was exposed to 12 specific ingredients. Ofloxacin was the most frequently exposed drug in the case and control groups, followed by ciprofloxacin and levofloxacin. In terms of the number of prescriptions, ofloxacin, ciprofloxacin, and levofloxacin were the most frequently prescribed drugs. The number and rate of prescriptions for each drug is shown in Table 2.

**Table 2:** Ingredients of the prescribed oral fluoroquinolones and numbers of prescribed subjects and episodes in the case and control groups.

Type of fluoroquinolone	Case group (n = 1,151)		Control group (n = 11,470)	
	Subjects (n = 146)	Prescriptions (n = 332)	Subjects (n = 1,384)	Prescriptions (n = 2,473)
Balofloxacin	3 (2.1%)	5 (1.5%)	16 (1.2%)	36 (1.2%)
Ciprofloxacin	42 (28.8%)	89 (26.8%)	339 (24.4%)	719 (23.5%)
Enoxacin	0 (0.0%)	0 (0.0%)	5 (0.4%)	10 (0.3%)
Fleroxacin	1 (0.7%)	10 (3.0%)	1 (0.1%)	2 (0.1%)
Gemifloxacin	1 (0.7%)	2 (0.6%)	2 (0.1%)	13 (0.4%)
Levofloxacin	37 (25.3%)	76 (22.9%)	349 (25.1%)	816 (26.6%)
Lomefloxacin	2 (1.4%)	4 (1.2%)	18 (1.3%)	37 (1.2%)
Moxifloxacin	1 (0.7%)	2 (0.6%)	6 (0.4%)	17 (0.6%)
Norfolxacin	6 (4.1%)	17 (5.1%)	63 (4.5%)	134 (4.4%)
Ofloxacin	49 (33.6%)	121 (36.5%)	569 (41.0%)	1228 (40.1%)
Tosufloxacin	4 (2.7%)	6 (1.8%)	13 (0.9%)	26 (0.9%)
Zabofloxacin	0 (0.0%)	0 (0.0%)	3 (0.2%)	14 (0.5%)

For analysis of the risk of retinal detachment according to exposure to oral FQ, a crude odds ratio of 1.06 (P = 0.53, 95% CI 0.88–1.28) was shown in the total group. In subgroups by exposure date, no subgroup showed statistical significance (all P’s>0.05). The significance of the adjusted odds ratio did not change in the total group (1.01, P = 0.96, 95% CI 0.81–1.24) or all subgroup (all P’s>0.05) when adjusted for age group at cohort entry, sex, household income, residual area, numbers of ophthalmologic visits and drug prescriptions during the year prior to the index date, diabetes, and degenerative myopia. Subgroup 3 (subjects exposed to FQ for 91–180 days before the index date) showed modest significance in adjusted odds ratio (1.42, P = 0.06, 95% CI 0.99–2.05). The adjusted odds ratio additionally adjusted for cataract surgery and vitrectomy (not for treatment of retinal detachment) was not significant in the total group (1.00, P = 0.99, 95% CI 0.81–1.24) and all subgroup (all P’s>0.05) (Table 3).

**Table 3:** Crude and adjusted odds ratios of the incidence of retinal detachment according to exposure to oral fluoroquinolone.

Exposure to oral fluoroquinolone during the year prior to the index date	Case (n = 1151)	Control (n = 11470)	Crude Odds Ratio (95% CI*)	Adjusted Odds Ratio^†^ (95% CI*)	Adjusted Odds Ratio^‡^ (95% CI*)
Non-exposed subjects	1005 (87.3%)	10081 (87.9%)	Reference	Reference	Reference
Exposed subjects	146 (12.7%)	1389(12.1)	1.06 (0.88–1.28)	1.01 (0.81–1.24)	1.00 (0.81–1.24)
0–14 days before the index date	13 (8.9%)	162(11.7)	0.81 (0.46–1.43)	0.77 (0.42–1.41)	0.75 (0.40–1.39)
14–90 days before the index date	37 (25.3%)	407(29.3)	0.92 (0.65–1.30)	0.82 (0.56–1.20)	0.80 (0.54–1.19)
91–180 days before the index date	42 (28.8%)	316(22.8)	1.35 (0.97–1.88)	1.42 (0.99–2.05)	1.39 (0.96–2.01)
181–270 days before the index date	30 (20.6%)	268(19.3)	1.13 (0.77–1.67)	1.09 (0.71–1.68)	1.11 (0.72–1.72)
271–365 days before the index date	24 (16.4%)	236(17.0)	1.03 (0.67–1.59)	0.91 (0.57–1.47)	0.92 (0.57–1.49)

* Confidence interval

† Adjusted by age group at cohort entry, sex, household income, residential area, numbers of ophthalmologic visits and drug prescriptions during the year prior to the index date, diabetes, and degenerative myopia

‡ Adjusted by age group at cohort entry, sex, household income, residential area, numbers of ophthalmologic visits and drug prescriptions during the year prior to the index date, diabetes, degenerative myopia, cataract surgery, glaucoma surgery, and keratoplasty, and vitrectomy not for the treatment of retinal detachment

## Discussion

According to the results of this study, exposure to oral FQ during the past year did not increase the risk of RRD based on data from a Korean database analyzed by a nested case-control design. There was only modest association in the subgroup with FQ exposure 91–180 days before the index date.

A nested case-control design was known as a useful tool for analyzing an event with low incidence in cohort [21] and time-dependent exposure [22] and was reported that it is less vulnerable to selection and time-dependent bias compared with simple case-control design [16]. Using a nested case-control design, we defined the visit to ophthalmologist as a definition of a nested cohort because it could include a total subject with a risk of RRD. In this study, an incidence sampling method was used. It is an essential method in a nested case-control design which selects matching control at each case outbreak time. By the mechanism of this method, a same subject could be a case or a matching control for other case at the different event time [21].

Unlike randomized clinical trials, there could be uncontrolled biases or confounders in observational studies using population-based data [18]. In studies associated with exposure to drugs, bias or confounders that were ignored by the investigators and critical for the results [17,18] might exist including time-related biases (immortal time bias and time-window bias), prevalent user bias, and confounding due to indication, generalizability, or exposure assessement [18].

Apart from the low significance of FQ on RRD in the clinical field and an unknown exact mechanism [23,24], control of biases was an important goal of this study. With the design of a nested case-control within a cohort, we could control for time-related biases. Using a cohort setting process, we attempted to achieve an equal distribution of baseline characteristics between the case and control groups. Adjustment for the confounders associated with health service utilization (numbers of ophthalmologic visits and prescriptions) had an effect on a bias, such as prevalent user bias. There was a limitation in exposure assessment because of the relatively short duration of FQ administration, various ingredients and doses of FQ used in this study, and selection of the most recent exposure. Because the significance of dose and duration of administration is not known, we might not be able to predict the effect of this limitation on the results.

Based on some reports [25–27], it is known that systemic FQ penetrates into the vitreal cavity within 6–8 hours. Theoretically, FQ could have an effect on the development of RRD by an unknown action. Previous studies have suggested that the risk of RD increased by 1.4–4.5 folds after exposure to oral FQ, especially for current users [6,7,9]. Based on the results of previous studies, it was suggested that the effect of FQ on the vitreoretinal surface might be an acute process.

Based on the background of acute action, we searched for FQ prescriptions in the year prior to the index date and used the most recent prescription as the date of exposure for subjects with multiple FQ prescriptions. However, this study showed a modest association between FQ and RRD in the 3–6 months after exposure, which is different from previous studies [6,7,9,28,29], even accounting for differences in study design and database. Differences in methodology and materials, ethnic differences in drug metabolism, and unknown confounders could explain the different results. In this study, the subgroup of current FQ users who were exposed to FQ for 2 weeks prior to the index date had a relatively smaller population (13 subjects in the case group) than other subgroups. The lack of sample subjects of current users might be not sufficient to prove the risk of FQ on RRD.

By the diagnostic code and surgical procedure code for RRD, it was nearly impossible to identify the exact onset time of RRD. All cases and matching controls in a nested cohort had an exam prior or equal to index date, but the method did not guarantee that all the RRD diagnosed on index date A case in a nested cohort could be an incident RRD, or an old, non-symptomatic RRD. This fundamental limitation of population based study could be a confounding factor for the analysis of odds ratio and latent period of RRD development.

There were some factors that limited the size of the case group. In consideration of the most likely mechanism for RRD associated with FQ [6,8,13], we excluded other types of retinal detachment that occurred due to exudative and tractional mechanisms which could be searched by ICD-10 codes of H33.2 (serous retinal detachment) and H33.4 (traction detachment of retina), respectively. In the KNHIS-NSC database, there was a critical weakness in the accuracy of diagnosis [30]. For validation of RRD diagnosis, only subjects who underwent scleral buckling or pars plana vitrectomy were included. Other procedures for treatment of RRD (cryopexy, pneumatic retinopexy, and barrier laser) were not included in the case definition because the EDI codes for pneumatic retinopexy (S5070, intravitreal injection) and barrier laser (S5160, retinal photocoagulation) were not specific to RRD. The limited case definition also had an effect on the defining of disease severity in the case group.

A limited number of confounding factors were used for adjustment. We did not include comorbidities not directly associated with RRD, such as hypertension, dyslipidemia, or renal disease. As mentioned above, the issue about the validation of diagnosis in the KNHIS-NSC data [30] also contributed to the limited number of confounding factors. The intraocular procedures of cataract surgery, glaucoma surgery, keratoplasty and vitrectomy were adjusted with considerable caution because the laterality of eye that underwent surgery (left or right) could not be identified by diagnostic or surgical codes.

As a fundamental limitation of studies using KNHIS-NSC data, uninsured drugs and procedures that are arbitrarily excluded from coverage by KNHIS cannot be identified in the KNHIS-NSC data [27]. Unlike intravitreal injection of insured drugs (such as ranibizumab), we could not search for intravitreal injections of uninsured drugs (such as bevacizumab). Medication adherence, which cannot be controlled in studies using big data [31], was also a limitation of this study.

Recently, FQ was reported to be associated with tendinopathy [23,32,33]. The pathophysiology of tendinopathy associated with FQ administration can be explained by FQ-associated collagen toxicity [34]. In previous studies [6,23], the association of RRD and oral FQ was explained by FQ-associated collagen toxicity due to its extended application. There could be more considerations for this extended application of pathophysiology because the majority of collagen fiber types were different between tendons (types I and III) [35] and the vitreoretinal interface (types II, IV, and VI) [36]. The lack of a mechanism to explain FQ-associated RRD might be the most important factor in the interpretation of this study and its clinical significance.

In conclusion, oral administration of FQ might not be associated with development of RRD using nationwide cohort database in Korea. For interpreting population-based studies, the status of bias control, especially time related bias in exposure assessment, should be prior to the result itself.

## Supporting information

S1 Table Specific components of fluoroquinolone and the first four digits of the main ingredient codes (Total Codes with 9 Digits) used to search for exposure to oral fluoroquinolone in the KNHIS-NSC (Korean National Health Insurance Service National Sample Cohort) 2002–2013. (DOCX)
