# Supplementary material for: Administration of oral fluoroquinolone and the risk of rhegmatogenous retinal detachment: A nationwide population-based study in Korea
Source: PLoS One. 2018 Apr 12;13(4):e0195563. doi: 10.1371/journal.pone.0195563 (PMC5896937; doi:10.1371/journal.pone.0195563)
Supplement: S1 Table — (DOCX) [file pone.0195563.s001.docx]

S1 Table. Specific Components of Fluoroquinolone and the First Four Digits of the Main Ingredient Codes (Total Codes with 9 Digits) Used to Search for Exposure to Oral Fluoroquinolone in the KNHIS-NSC (Korean National Health Insurance Service National Sample Cohort) 2002-2013

| Ingredient | First four digits  of the main ingredient code* |
| --- | --- |
| Ciprofloxacin | 1341 |
| Levofloxacin | 1832, 1849 |
| Moxifloxacin | 3803 |
| Lomefloxacin | 1849 |
| Norfolxacin | 2033 |
| Ofloxacin | 2039 |
| Tosufloxacin | 2422 |
| Balofloxacin | 4289 |
| Gemifloxacin | 4429 |
| Zabofloxacin | 1371 |
| Enoxacin | 1520 |
| Fleroxacin | 1594 |
| Grepafloxacin | 1674 |
| Pefloxacin | 2095 |
| Rosofloxacin | 3804 |
| Rufloxacin | 3588 |
| Sparfloxacin | 2307 |
| Gatifloxacin | 4348 |

* Only oral drugs were selected and were filtered by the seventh digit (digit for the administration method), and then the ingredients of fluoroquinolone were searched by the first four digits (digits for ingredients). The code for dosage (5-6th digits) and dosage form (8-9th digits) were not used in the search for exposure.
